# Supplementary material for: Effect of Self-Management Support for Elderly People Post-Stroke: A Systematic Review
Source: Geriatrics (Basel). 2020 Jun 18;5(2):38. doi: 10.3390/geriatrics5020038 (PMC7345508; doi:10.3390/geriatrics5020038)
Supplement: Supplementary file 1 [file geriatrics-05-00038-s001.pdf]

Supplementary material

Table S1: PRISMA 2009 checklist

Section/topic	#	Checklist item	Reported on page #
Title	1	Identify the report as a systematic review, meta-analysis, or both.	1
**ABSTRACT**			
Structured summary	2	Provide a structured summary including, as applicable: background; objectives; data sources; study eligibility criteria, participants, and interventions; study appraisal and synthesis methods; results; limitations; conclusions and implications of key findings; systematic review registration number.	1
**INTRODUCTION**			
Rationale	3	Describe the rationale for the review in the context of what is already known.	3
Objectives	4	Provide an explicit statement of questions being addressed with reference to participants, interventions, comparisons, outcomes, and study design (PICOS).	4
**METHODS**			
Protocol and registration	5	Indicate if a review protocol exists, if and where it can be accessed (e.g., Web address), and, if available, provide registration information including registration number.	4
Eligibility criteria	6	Specify study characteristics (e.g., PICOS, length of follow-up) and report characteristics (e.g., years considered, language, publication status) used as criteria for eligibility, giving rationale.	4
Information sources	7	Describe all information sources (e.g., databases with dates of coverage, contact with study authors to identify additional studies) in the search and date last searched.	5
Search	8	Present full electronic search strategy for at least one database, including any limits used, such that it could be repeated.	Table S2
Study selection	9	State the process for selecting studies (i.e., screening, eligibility, included in systematic review, and, if applicable, included in the meta-analysis).	6

Data collection process	10	Describe method of data extraction from reports (e.g., piloted forms, independently, in duplicate) and any processes for obtaining and confirming data from investigators.	6
Data items	11	List and define all variables for which data were sought (e.g., PICOS, funding sources) and any assumptions and simplifications made.	5
Risk of bias in individual studies	12	Describe methods used for assessing risk of bias of individual studies (including specification of whether this was done at the study or outcome level), and how this information is to be used in any data synthesis.	6
Summary measures	13	State the principal summary measures (e.g., risk ratio, difference in means).	N/A
Synthesis of results	14	Describe the methods of handling data and combining results of studies, if done, including measures of consistency (e.g., I^2) for each meta-analysis.	6
Risk of bias across studies	15	Specify any assessment of risk of bias that may affect the cumulative evidence (e.g., publication bias, selective reporting within studies).	N/A
Additional analyses	16	Describe methods of additional analyses (e.g., sensitivity or subgroup analyses, meta-regression), if done, indicating which were pre-specified.	N/A
**RESULTS**			
Study selection	17	Give numbers of studies screened, assessed for eligibility, and included in the review, with reasons for exclusions at each stage, ideally with a flow diagram.	7
Study characteristics	18	For each study, present characteristics for which data were extracted (e.g., study size, PICOS, follow-up period) and provide the citations.	8
Risk of bias within studies	19	Present data on risk of bias of each study and, if available, any outcome level assessment (see item 12).	15 + Table S4
Results of individual studies	20	For all outcomes considered (benefits or harms), present, for each study: (a) simple summary data for each intervention group (b) effect estimates and confidence intervals, ideally with a forest plot.	16
Synthesis of results	21	Present results of each meta-analysis done, including confidence intervals and measures of consistency.	N/A
Risk of bias across studies	22	Present results of any assessment of risk of bias across studies (see Item 15).	N/A
Additional analysis	23	Give results of additional analyses, if done (e.g., sensitivity or subgroup analyses, meta-regression [see Item 16]).	N/A
**DISCUSSION**			

Summary of evidence	24	Summarize the main findings including the strength of evidence for each main outcome; consider their relevance to key groups (e.g., healthcare providers, users, and policy makers).	20
Limitations	25	Discuss limitations at study and outcome level (e.g., risk of bias), and at review-level (e.g., incomplete retrieval of identified research, reporting bias).	21
Conclusions	26	Provide a general interpretation of the results in the context of other evidence, and implications for future research.	22
**FUNDING**			
Funding	27	Describe sources of funding for the systematic review and other support (e.g., supply of data); role of funders for the systematic review.	24

From: Moher D, Liberati A, Tetzlaff J, Altman DG, The PRISMA Group (2009). Preferred Reporting Items for Systematic Reviews and Meta-Analyses: The PRISMA Statement. PLoS Med 6(7): e1000097. doi:10.1371/journal.pmed1000097

For more information, visit: **www.prisma-statement.org**.

Table S2: Electronic search strategies

Search strategies for literature in three databases on 28th May 2019: PubMed (1966 to May 2019), Embase (1980 to May 2019) and PsycInfo (1967 to May 2019). A follow-up search in the three databases was added on 29th April 2020

28/5/2019 Advanced search - PubMed - NCBI
Search **(((((((("Stroke Rehabilitation"[Mesh) OR "Stroke"[Mesh]) OR stroke[Text Word])) AND**
((((((("Self Care"[Mesh]) OR "Self Efficacy"[Mesh]) OR "Patient Participation"[Mesh]) OR self
car*[Text Word]) OR self manag*[Text Word]) OR self efficac*[Text Word]) OR "Personal
Autonomy"[Mesh]) OR autonomy[Text Word]))) AND ((((((("Controlled Clinical Trial" [Publication
Type]) OR randomized[Title/Abstract]) OR randomly[Title/Abstract]) OR trial[Title/Abstract]) OR
single blind*[Title/Abstract]) OR double blind*[Title/Abstract]) OR ((("Single-Blind Method"
[Mesh]) OR "Double-Blind Method"[Mesh]) OR "Random Allocation"[Mesh]))) AND ((Danish[lang] OR English[lang] OR Norwegian[lang] OR Swedish[lang])))

Database: Embase <1974 to 2019 May 28>
Search Strategy:
----
1 stroke rehabilitation/ (2433)
2 exp cerebrovascular accident/ (187708)
3 stroke.ab,kw,ti. (356484)
4 or/1-3 (411417)
5 exp self care/ (74992)
6 self concept/ (83395)
7 patient participation/ (25265)
8 "self car*".ab,kw,ti. (23748)
9 "self manag*".ab,kw,ti. (25849)
10 "self efficac*".ab,kw,ti. (30008)
11 personal autonomy/ (12987)
12 autonomy.ab,kw,ti. (33536)
13 or/5-12 (236873)
14 4 and 13 (3910)
15 exp controlled clinical trial/ (734794)
16 double blind procedure/ or single blind procedure/ (193974)
17 randomized.ab,ti. (683459)
18 randomly.ab,ti. (410323)
19 trial.ab,ti. (775077)
20 "single blind*".ab,ti. (22924)

21 "double blind*".ab,ti. (197817)
22 or/15-21 (1762996)
23 14 and 22 (836)
24 limit 23 to (conference abstract or conference paper or "conference review" or editorial or letter) (271)
25 23 not 24 (565)
26 limit 25 to (danish or english or norwegian or swedish) (532)

Database: PsycINFO <1806 to May Week 3 2019>
Search Strategy:

----

1 cerebrovascular accidents/ (19743)
2 stroke.ab,id,ti. (31535)
3 1 or 2 (33095)
4 self-determination/ (4237)
5 self-management/ (5991)
6 self-care skills/ (4278)
7 empowerment/ (7105)
8 "independence (personality)"/ (4994)
9 self-efficacy/ (21103)
10 client participation/ (1957)
11 "self car*".ab,id,ti. (9046)
12 "self manag*".ab,id,ti. (9311)
13 "self efficac*".ab,id,ti. (37656)
14 autonomy.ab,id,ti. (28531)
15 or/4-14 (98737)
16 3 and 15 (620)
17 clinical trials/ (11329)
18 randomized.ab,ti. (67723)
19 randomly.ab,ti. (68919)
20 trial.ab,ti. (98669)
21 "single blind*".ab,ti. (2277)
22 "double blind*".ab,ti. (22835)
23 or/17-22 (199402)
24 16 and 23 (108)
25 limit 24 to (danish or english or norwegian or swedish) (108)

Table S3: Overview of measurements

Psychosocial outcome measures	Measurements (Authors, year)	Pooled number of participants (Studies)
Self-Management	• Stroke Self-Management Outcome Expectation Scale (Lo et al., 2018)<br>• Stroke Self-Management Behaviors Performance Scale (Lo et al. 2018)<br>• 9 items from The Chinese Self-Management Behavior Questionnaire (Sit et al. 2016)	338 (2)
Self-Efficacy	• 10 questions that assessed the patient's recovery self-efficacy (Glass et al., 2004)<br>• Self-efficacy Scale (Kendall et al., 2006)<br>• Stroke Self-Efficacy Questionnaire (Lo et al., 2018)<br>• 6 items from The Chinese Self-Management Behavior Questionnaire (Sit et al., 2016)	729 (4)
Quality of Life	• Stroke Adapted 30-item Sickness Impact Profile (Allen et al., 2002)<br>• Stroke Specific Quality of Life scale (Allen et al., 2009; Kendall et al., 2006)<br>• Short From 36 Physical Component Summary (Fu et al., 2020)<br>• Short Form 12 Physical Component Summary (Fu et al., 2020)<br>• European Quality of Life-5 Dimensions-5 levels (Fu et al., 2020)<br>• A five-level, single-item global rating scale (Glass et al., 2004)<br>• Stroke and Aphasia Quality of Life Scale-39 (Hjelle et al., 2019)	1589 (6)
Depression	• Center for Epidemiologic Studies Depression Scale (Allen et al., 2002; Allen et al.; 2009; Glass et al., 2004)<br>• 13-item Geriatric Depression Scale Short Form (Bishop et al., 2015)<br>• Yale-Brown Single-item Questionnaire (Hjelle et al., 2019)	1138 (5)
Activities of Daily Living	• Stroke Impact Scale: Subscale 5 regarding perceived difficulties in activities of daily living (Guidetti & Ytterberg, 2010)<br>• Occupational Gaps Questionnaire (Guidetti & Ytterberg, 2010)<br>• Chinese Lawton Instrumental Activities of Daily Living Scale (Sit et al., 2016)	250 (2)
Active Lifestyle	• An investigator-generated questionnaire measuring Stroke Knowledge and Lifestyle Modification (Allen et al., 2009)<br>• Combining the scores on five timed tests of functional capacity, including writing a sentence, simulated eating, simulated dressing, turning in a circle, and walking 20 feet (Glass et al., 2004)<br>• A description of stage of change in relation to risk factors identified by the patient, including exercise (none; low:10-15 minutes/1-2x/week; moderate: 15-30 minutes/3-4x/week; high: 30+ minutes/5-7x/week) (Green et al., 2007)	871 (3)
Other Measures	• Frenchay Activities Index (Bishop et al., 2015; Fu et al., 2020; Guidetti & Ytterberg, 2010)<br>• Family Assessment Device (Bishop et al., 2015)<br>• Perceived Criticism Scale (Bishop 2015)<br>• Caregiver Strain Index (Fu et al., 2020)<br>• A modified version of Barrera's Inventory of Socially Supportive Behaviors (Glass et al., 2004)<br>• A description of stage of change in relation to risk factors identified by the patient, including stress (none, mild, moderate, high) (Green et al., 2007)<br>• Stroke Impact Scale: Subscale 8 regarding perceived difficulties in participation (Guidetti & Ytterberg, 2010)<br>• Life Satisfaction Scale 11 (Guidetti & Ytterberg, 2010)	FAI[1]: 489 (3)<br>FAD[2]: 49 (1)<br>PCS[3]: 49 (1)<br>CSI[4]: 400 (1)<br>ISSB[5]: 291 (1)<br>Stress[6]: 200 (1)<br>SIS[7]: 40 (1)<br>LiSat-11[8]: 40 (1)<br>GHQ-28[9]: 322 (1)<br>SOC-13[10]: 322 (1)

- General Health Questionnaire-28 (Hjelle et al., 2019)
- Sense of Coherence Scale-13 (Hjelle et al., 2019)

[1]FAI = Frenchay Activities Index; [2] FAD = Family Assessment Device; [3]PCS = Perceived Criticism Scale; [4]CSI = Caregiver Strain Index; [5] ISSB = A modified version of Barrera's Inventory of Socially Supportive Behaviors; [6]Stress = A description of stage of change in relation to stress; SIS[7] = Stroke Impact Scale; [8]LiSat-11 = Life Satisfaction Scale 11; [9]GHQ-28 = General Health Questionnaire-28; [10]SOC-13 = Sense of Coherence Scale-13

Table S4: An overview of the answers to signalling questions, together with free-text justification of the answers

Domain 1: Risk of bias arising from the randomization process

Author (year)	Signalling question			Domain-level judgement	
	**1.1 Sequence random?**	**1.2 Allocation concealed?**	**1.3 Imbalance suggest problem?**	**Default risk of bias**	**Remarks**
Allen et al. (2002)	PY	PY	N	Low	Sound randomization methods and baseline balance
Allen et al. (2009)	PY	PY	NI	Low	Sound randomization methods and baseline balance
Bishop et al. (2015)	PY	PY	PN	Low	Sound randomization methods and baseline balance
Fu et al. (2020)	Y	Y	N	Low	Sound randomization methods and baseline balance
Glass et al. (2004)	Y	PY	PY	Some concern	Sound randomization methods. Fewer participant in the control group were depressed at baseline.
Green et al. (2007)	PY	Y	N	Low	Sound randomization methods and baseline balance
Guidetti & Ytterberg (2010)	Y	Y	PN	Low	Sound randomization methods and baseline balance
Hjelle et al. (2019)	Y	Y	PY	Some concern	Sound randomization methods. Fewer participant in the interventionl group were depressed at baseline.
Kendall et al. (2006)	PY	Y	NI	Low	Sound randomization methods and baseline balance
Lo et al. (2018)	Y	Y	PN	Low	Sound randomization methods and baseline balance
Sit et al. (2016)	Y	Y	N	Low	Sound randomization methods and baseline balance

Y/PY = ‘Yes’ or ‘Probably yes’; N/PN = ‘No’ or ‘Probably no’; NI = ‘No information’

Domain 2: Risk of bias due to deviations from the intended interventions (*effect of assignment to intervention)*

**Author (year)**	**Signalling question**					**Domain-level judgement**	
	Part 1: Questions 2.1 to 2.5						
	**2.1 Participants aware?**	**2.2 Personnel aware?**	**2.3 Any deviations?**	**2.4 Affecting outcomes?**	**2.5 Balanced deviations?**	**Default risk of bias for part 1**	**Remarks**
Allen et al. (2002)	Y	PY	PN	---	---	Low	It was not possible to blind participants, and people delivering the intervention. However, nothing unexpected seemed to occur in the implementation of the intervention
Allen et al. (2009)	NI	NI	Y	N	---	Some concern	No information about blinding, however it is assumed that blinding was not possible. Furthermore, little time was devoted to addressing health or psychosocial issues as intended
Bishop et al. (2015)	NI	NI	PN	---	---	Low	No information about blinding, however it is assumed that blinding was not possible. Nothing unexpected seemed to occur in the implementation of the intervention
Fu et al. (2020)	PY	PY	N	---	---	Low	No information about blinding, however it is assumed that blinding was not possible. Nothing unexpected seemed to occur in the implementation of the intervention
Glass et al. (2004)	Y	Y	N	---	---	Low	Participants and interventionists were aware of the patient's treatment assignment. However, nothing unexpected occurred in the implementation of the intervention
Green et al. (2007)	PY	Y	N	---	---	Low	Blinding to study group was not possible. However, nothing

							unexpected occurred in the implementation of the intervention
Guidetti & Ytterberg (2010)	PY	PY	Y	Y	PY	High	No information about blinding, however it is assumed that blinding was not possible. High probability of spill-over effect from the intervention to participants in the control group
Hjelle et al. (2019)	Y	Y	N	---	---	Low	Group allocations were communicated to the patient and the people delivering the intervention. However, nothing unexpected occurred in the implementation of the intervention
Kendall et al. (2006)	PY	PY	NI	---	---	Some concern	No information about blinding, however it is assumed that blinding was not possible. No information about whether deviations arose because of the trial context
Lo et al. (2018)	PY	PY	NI	---	---	Some concern	No information about blinding, however it is assumed that blinding was not possible. No information about whether deviations arose because of the trial context
Sit et al. (2016)	PY	PY	NI	---	---	Some concern	No information about blinding, however it is assumed that blinding was not possible. No information about whether deviations arose because of the trial context
	Part 2: Questions 2.6 and 2.7						
	**2.6 Appropriate analysis?**		**2.7 Potential impact on result due to switching groups in analysis?**			**Default risk of bias for part 2**	**Remarks**
Allen et al. (2002)	NI		PN			Some concern	Missing information about analysis used to estimate the effect of assignment to intervention

Allen et al. (2009)	Y	---	Low	Used intention-to-treat analysis.
Bishop et al. (2015)	Y	---	Low	Used intention-to-treat analysis
Fu et al. (2020)	Y	---	Low	They do not mention whether they used intention-to-treat analysis, but that is what they did according to Figure 1
Glass et al. (2004)	PY	---	Low	Intention-to-treat analysis was used for Barthel Index. It is assumed that intention-to-treat analysis is used for the other outcomes also
Green et al. (2007)	Y	---	Low	Used intention-to-treat analysis
Guidetti & Ytterberg (2010)	Y	---	Low	Used intention-to-treat analysis
Hjelle et al. (2019)	Y	---	Low	Used intention-to-treat analysis
Kendall et al. (2006)	NI	PN	Some concern	Missing information about analysis used to estimate the effect of assignment to intervention
Lo et al. (2018)	Y	---	Low	Used intention-to-treat analysis
Sit et al. (2016)	Y	---	Low	Used intention-to-treat analysis
**Criteria for the domain**				
‘Low’ risk of bias in Part 1 AND ‘Low’ risk of bias in Part 2			Low	
‘Some concerns’ in either Part 1 OR in Part 2, AND NOT ‘High’ risk in either part			Some concern	
‘High’ risk of bias in in either Part 1 OR in Part 2			High	

Y/PY = ‘Yes’ or ‘Probably yes’; N/PN = ‘No’ or ‘Probably no’; NI = ‘No information’

Domain 3: Risk of bias due to missing outcome data

Author (year)	Signalling question				Domain-level judgement	
	3.1 Complete data?	3.2 Evidence of no bias?	3.3 Could depend on true?	3.4 Likely depend on true?	Default risk of bias	Remarks
Allen et al. (2002)	NI	PN	NI	NI	High	Insufficient information about reasons for dropout. Does not differentiate between the dropout rate in the two groups. Total dropout = 21%. No information about methods correcting for missing outcome data.
Allen et al. (2009)	N	N	NI	PN	Some concern	Dropouts: 13% (Intervention group), 19% (control group. Do not describe reasons for dropout. Sensitivity analyses was not performed.
Bishop et al. (2015)	N	N	Y	PN	Some concern	Does not differentiate between the dropout rate in the two groups. Total dropout = 16% (stroke individuals) and 22% (caregivers). No flowchart. No information about methods correcting for missing outcome data.
Fu et al. (2020)	Y	---	---	---	Low	Dropouts: 7% (TC 1), 4% (TC 2) and 4% (Control group). The reasons for dropout were more or less similar in the three groups. Sensitivity analyses was made.
Glass et al. (2004)	Y	---	---	---	Low	Dropouts: 8% (interventions group) and 10% (control group). The reasons for dropout are similar in the two groups. Sensitivity analyses was made for Barthel Index. It is assumed that this also applies to the other outcomes.
Green et al. (2007)	N	N	Y	Y	High	Uneven dropouts: 28% (intervention group), 8% (control group). 20% voluntarily discontinued participation in the intervention group. Sensitivity

						analyses was not performed.
Guidetti & Ytterberg (2010)	N	N	Y	Y	High	Uneven dropouts: 47% (intervention group), 33% (control group). Dropout caregivers: 38%. Some participants said it was too strenuous to participate. Used 'last-observation-carried-forward' to correct for bias due to missing outcome data
Hjelle et al. (2019)	Y*	PN	Y	PY	Some concern	Dropouts: 7% (intervention group) and 4% (Control group). Small dropout, but the dropout rate was greatest in the intervention group and connected to group allocation Used multiple imputations.
Kendall et al. (2006)	N	PN	Y	PN	Some concern	Does not differentiate between the dropout rate in the two groups. Total dropout = 29%. No flowchart. Sensitivity analyses was not performed
Lo et al. (2018)	N	N	Y	Y	High	Dropouts: 19% (intervention group), 14% (control group). 38% of the participant in the intervention group received all sessions. Sensitivity analyses was not performed. Compared with per-protocol
Sit et al. (2016)	N	N	Y	PN	Some concern	Uneven dropout: 11% (intervention group), 22% (control group). Sensitivity analyses was not performed

Y/PY = 'Yes' or 'Probably yes'; N/PN = 'No' or 'Probably no'; NI = 'No information'

***** The other signalling questions were also assessed as they seem to be important in relation to the judgement

Domain 4: Risk of bias in measurement of the outcome

Author (year)	Signalling question					Domain-level judgement	
	**4.1 Inappropriate?**	**4.2 Differed between groups?**	**4.3 Aware?**	**4.4 Could be influenced?**	**4.5 Likely to be influenced?**	**Default risk of bias for**	**Remarks**
Allen et al. (2002)	N	PN	Y	PY	PN	Some concern	Participant-reported outcomes = the outcome assessor was not blinded, as it was impossible to blind the participants to group assignment
Allen et al. (2009)	PN**	N	PY	PY	PN	Some concern	The investigator-generated questionnaire measuring present activity may not be sufficiently sensitive and validated. Participant-reported outcomes = the outcome assessor was not blinded, as it was impossible to blind the participants to group assignment
Bishop et al. (2015)	N	PN	PY	PY	PN	Some concern	Participant-reported outcomes = the outcome assessor was not blinded, as it was impossible to blind the participants to group assignment
Fu et al. (2020)	N	N	PY	PY	PN	Some concern	Participant-reported outcomes = the outcome assessor was not blinded, as it was impossible to blind the participants to group assignment
Glass et al. (2004)	PY***	(PN)	(PY)	(PY)	(PN)	High	The methods used to measure quality of life, physical performance and self-efficacy may not be sufficiently sensitive and validated. Furthermore, it was primarily participant-reported

							outcomes = the outcome assessor was not blinded, as it was impossible to blind the participants to group assignment
Green et al. (2007)	PY	---	---	---	---	High	The psychosocial measurements may not be sufficiently sensitive and validated.
Guidetti & Ytterberg (2010)	N	N	PY	PY	PN	Some concern	Participant-reported outcomes = the outcome assessor was not blinded, as it was impossible to blind the participants to group assignment
Hjelle et al. (2019)	N	PY	Y	PY	PN	Some concern	Participant-reported outcomes = the outcome assessor was not blinded, as it was impossible to blind the participants to group assignment
Kendall et al. (2006)	N	N	NI	PY	PN	Some concern	Blinding was not reported. However, participant-reported outcomes = the outcome assessor was not blinded, as it was impossible to blind the participants to group assignment
Lo et al. (2018)	N	N	PY	PY	PN	Some concern	Participant-reported outcomes = the outcome assessor was not blinded, as it was impossible to blind the participants to group assignment
Sit et al. (2016)	PN	PN	PY	PY	PN	Some concern	Participant-reported outcomes = the outcome assessor was not blinded, as it was impossible to blind the participants to group assignment

Y/PY = ‘Yes’ or ‘Probably yes’; N/PN = ‘No’ or ‘Probably no’; NI = ‘No information’

**One of three psychosocial measurements may not be sufficiently sensitive and validated

*** Not all psychosocial measurements were subject to uncertainty regarding sensitivity and validity, which is why the other signalling questions also were assessed

Domain 5: Risk of bias in selection of the reported result

Author (year)	Signalling question			Domain-level judgement	
	**5.1 In accordance with plan?**	**5.2 Selected from multiple outcomes?**	**5.3 Selected from multiple analyses?**	**Default risk of bias**	**Remarks**
Allen et al. (2002)	NI	NI	NI	Some concern	No information about pre-specified analysis intentions
Allen et al. (2009)	Y	N	N	Low	Data are analysed in accordance with pre-specified intentions
Bishop et al. (2015)	PN	NI	NI	Some concern	Insufficient information about pre-specified analysis intentions
Fu et al. (2020)	Y	N	N	Low	Data are analysed in accordance with pre-specified intentions
Glass et al. (2004)	Y	Y	N	High	Data form Barthel Index are analysed in accordance with pre-specified intentions. However, the psychosocial outcomes are missing or insufficient reported
Green et al. (2007)	PN	PN	PN	Some concern	Insufficient information about pre-specified analysis intentions. Investigators defined stress as a lifestyle risk factor. However, information about stress are insufficient reported
Guidetti & Ytterberg (2010)	NI	NI	NI	Some concern	No information about pre-specified analysis intentions. However, it is a feasibility study and could be perceived as a pre-study
Hjelle et al. (2019)	Y	N	N	Low	Data are analysed in accordance with pre-specified intentions
Kendall et al. (2006)	NI	PY	PN	High	No information about pre-specified analysis intentions. Quality of life is overreported in relation to self-efficacy, and the different areas of quality of life is unevenly reported
Lo et al. (2018)	N	NI	NI	Some concern	Insufficient information about pre-

					specified analysis intentions. Do not report health-related quality of life, depressive symptoms and community reintegration as mentioned in the protocol
Sit et al. (2016)	NI	N	N	Some concern	Insufficient information about pre-specified analysis intentions

Y/PY = ‘Yes’ or ‘Probably yes’; N/PN = ‘No’ or ‘Probably no’; NI = ‘No information’
